# Genomics-directed activation of cryptic natural product pathways deciphers codes for biosynthesis and molecular function

**DOI:** 10.1007/s11418-020-01466-x

**Published:** 2020-12-03

**Authors:** Yuta Tsunematsu

**Affiliations:** Department of Pharmaceutical Sciences, University of Shizuoka, Shizuoka, 422-8526 Japan

**Keywords:** Biosynthesis, Natural products, Spiro compounds

## Abstract

Natural products, which can be isolated from living organisms worldwide, have played a pivotal role in drug discovery since ancient times. However, it has become more challenging to identify a structurally novel molecule with promising biological activity for pharmaceutical development, mainly due to the limited methodologies for their acquisition. In this review, we summarize our recent studies that activate the biosynthetic potential of filamentous fungi by genetic engineering to harness the metabolic flow for the efficient production of unprecedented natural products. The recent revolution in genome sequencing technology enables the accumulation of vast amounts of information on biosynthetic genes, the blueprint of the molecular construction. Utilizing the established heterologous expression system, activation of the pathway-specific transcription factor coupled with a knockout strategy, and manipulating the global regulatory gene, the biosynthetic genes were exploited to activate biosynthetic pathways and decipher the encoded enzyme functions. We show that this methodology was beneficial for acquiring fungal treasures for drug discovery. These studies also enabled the investigation of the molecular function of natural products in fungal development.

## Introduction

Humans utilize natural products, defined as small organic molecules, originating from natural resources such as plants, animals, and microbes [1]. The prescription of willow tree extract for the treatment of pain, fever, and childbirth by Hippocrates of Kos, the father of all doctors, more than 2000 years ago is an example of nature-driven drug discovery [2]. Although these medications were administered as crude materials since ancient times, the active substance salicin was identified and isolated for the first time in the nineteenth century. Purification of the substance enabled the development of acetylsalicylic acid, aspirin, a painkiller used to reduce pain, fever, and inflammation. Historically, once a compound was isolated as a pure material from natural resources, analysis of its effects on living organisms was greatly accelerated, making it possible to develop novel medicines. Therefore, human beings have been searching for compounds exhibiting useful biological activities from natural resources worldwide since the nineteenth century. At present, no small number of natural products or artificially designed molecules, inspired by the structure of natural products are being used as drugs [3]. Additionally, they are essential as medicines and research tools for unveiling many biological phenomena. For instance, leptomycin B, which was initially identified as an antifungal agent from the soil bacterium *Streptomyces* sp. [4], has revealed the function of its molecular target, Crm1, as a protein responsible for nuclear transportation in eukaryotes [5, 6]. Today, the molecule is commercially available and used in various biochemical studies such as investigations on site-specific roles of proteins that are mobile through the nuclear membrane. Moreover, the association between cancer and Crm1 has attracted attention, and an alternative Crm1 inhibitor, KPT-330 (Selinexor) [7], has been developed for the treatment of refractory myeloma. In addition, there are many examples of natural products that significantly contribute to opening a door for cryptic biological phenomena. As mentioned above, discovering novel natural products remains meaningful from the insights of both academic and industrial perspectives.

However, it is not easy to find new bioactive natural products using traditional methods [8]. Historically, to effectively identify unique substances, the biological sources searched for natural products have successfully shifted from readily available terrestrial plants to microorganisms (especially actinomycetes and molds) and then to living organisms at places not easily accessible by people, such as deep sea or extreme environments. Therefore, most organisms on Earth have been targeted as sources, with the most easily accessible substances already discovered. Indeed, many pharmaceutical companies that previously established their own natural product-based drug discovery departments and conducted research around the 1980s have since closed down. The focus has shifted to alternative modalities, such as antibodies, oligonucleotides, and regenerative medicines. Of the reasons that could be considered are (1) the discovery of new compounds is more difficult than before and (2) the identification of active compounds, structure determination, structure optimization, and establishment of a sustainable supply system are time-consuming and resource intensive.

In contrast, the revolution in next-generation sequencing technologies in the early twenty-first century sheds light on the revival of natural product-based drug discovery. Low costs and rapid deciphering of microbial genomes and transcriptomes, coupled with advances in genetic engineering technology, have revealed numerous natural product biosynthetic pathways [9]. They reported that structurally complicated natural products are constructed from simple building units such as amino acids and organic acids, so-called primary metabolites, through successive biochemical reactions carried out by multiple enzymes. Therefore, the accumulation of information on various biosynthetic enzymes has made it possible to utilize the biosynthetic genes discovered in the genome sequences as a blueprint for the construction of natural product scaffolds.

In particular, it has become possible to predict the molecular functions of biosynthetic enzymes, such as polyketide synthases (PKSs) [10] and non-ribosomal peptide synthetases (NRPSs) [11] which are responsible for the construction of scaffolds for natural products based on the components of domain architecture. Furthermore, phylogenetic analysis and insight into the conserved catalytic residues retrieved from the amino acid sequence of the enzymes provide information about the catalytic processes [12]. Alternatively, the DNA sequence analysis now allows us to estimate which genes are likely to lead to a novel natural product.

Fortunately, many studies have shown that microbial genomes contain more orphan biosynthetic genes than the number of compounds identified so far. For example, the genome of the filamentous fungus *Aspergillus terreus*, which is the producer of lovastatin, a treatment for dyslipidemia, contains more than 50 biosynthetic gene clusters (BGCs), many of which have unknown functions [13]. Moreover, transcriptome analysis revealed that a large number of biosynthetic genes are transcriptionally silent under conventional laboratory culture conditions, indicating that they are cryptic and unexploited but have the potential to produce molecules with unprecedented chemical structures [14]. With this background, we focused on the silent genes encoded in the fungal genome and sought to engineer the genes to activate the hidden biosynthetic pathways to produce structurally novel metabolites effectively. Additionally, en route to the aim, we have encountered nature-driven fascinating chemistry prompted by enzyme catalysis, which is also introduced and discussed in this review article.

## Engineered biosynthesis of spirotryprostatin

**Fig. 1:**
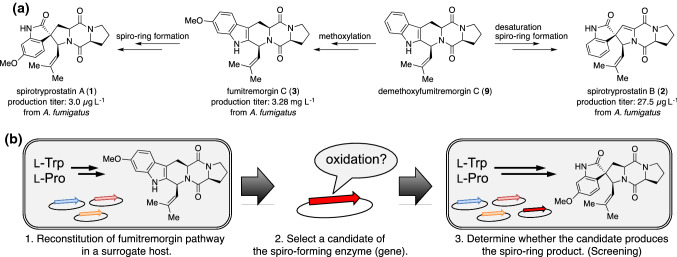
Spirotryprostatins biosynthesis. **a** Predicted biosynthetic pathway toward spirotryprostatins A and B. **b** The strategy we applied for the investigation of the spiro-forming enzyme

The natural products we initially targeted to be produced by genetic engineering-based activation of the pathway in a microbe were spirotryprostatins A (**1**) and B (**2**), originally isolated from the filamentous fungus *Aspergillus fumigatus* BM939 with extremely low titers (3.0 µg L^−1^ for **1** and 27.5 µg L^−1^ for **2**) [15]. The compounds were reported to show cell cycle arrest in the mouse breast cancer cells tsFT210 at modest concentrations. Additionally, their complex structures have attracted attention in synthetic chemistry, and achievements of the total synthesis, including the elegant construction of the spiro ring, were reported by several groups [16, 17]. Alternatively, it remains unclear how the microbes build up the spirocycle in their metabolic process. We considered that the drastically decreased production titer of **1** and **2** in *A. fumigatus* as compared to the related compounds [for example, the titer of fumitremorgin C (**3**): 3.28 mg L^−1^] were likely caused by the transcriptionally silent biosynthetic gene that is responsible for spiro ring formation (Fig. 1a). Therefore, we hypothesized that the overexpression of biosynthetic genes will result in the overproduction of molecules.

Our initial attempt to identify the gene(s) responsible for the formation of the spiro ring using a gene-targeting approach was ineffective due to the unobservable productions of **1** and **2** in the cultured *A. fumigatus* A1159 by LC–MS analysis. This indicated that the reverse genetic strategy was not applicable to sufficiently identify the gene that yields the trace constituents. Therefore, to tackle this obstacle, we employed an alternative approach based on heterologous expression of the biosynthetic genes of the spirotryprostatin pathway in a surrogate host. We hypothesized that this strategy would characterize the spiro-forming enzyme and provide a sustainable production system for the molecules.

The strategy for screening the spiro-ring-forming enzyme is as follows (Fig. 1b). First, the biosynthetic pathway of fumitremorgin C (**3)**, a plausible precursor of **1**, is reconstituted in a heterologous host such as *Saccharomyces cerevisiae* through the introduction and overexpression of biosynthetic genes of **3**.[18] Second, a candidate gene that plausibly codes for the enzyme catalyzing the desired reaction is introduced into the host with the fumitremorgin pathway. The production of **1** and **2** in the heterologous host allowed us to investigate the screened gene’s activity as a spiro-ring-forming enzyme.

**Fig. 2:**
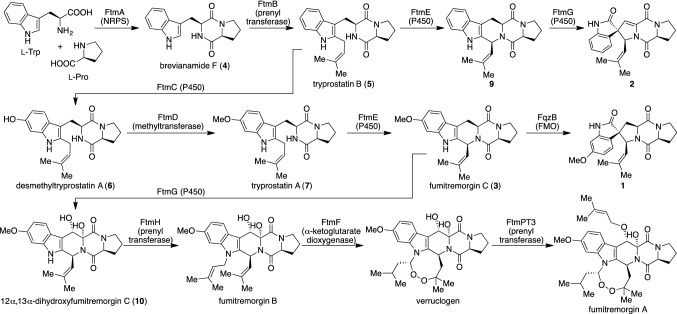
The biosynthetic pathway of tryprostatin/spirotryprostatin/fumitremorgin-class fungal metabolites

**Fig. 3:**
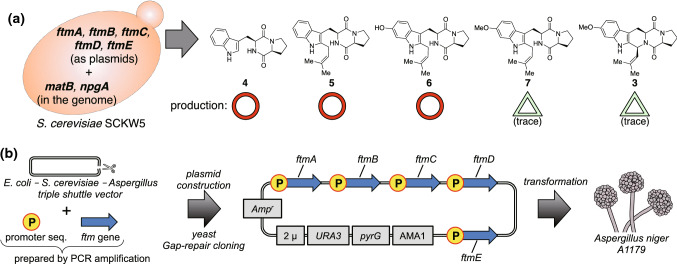
Reconstitution of the biosynthetic pathway in the surrogate host. **a** *S. cerevisiae*-based heterologous expression system for producing tryprostatins. **b** *A. niger*-based heterologous expression system

Based on the reported biosynthetic pathway of **3** [19–22], five biosynthetic enzymes are required for the production of **3** (Fig. 2). As the initial step, FtmA, which is a non-ribosomal peptide synthetase, catalyzes the formation of brevianamide F (**4**) from l-tryptophan and l-proline. Then, a prenyltransferase FtmB provides tryprostatin B (**5**) by adding a prenyl group to the indole ring of **4**. Subsequent aromatic hydroxylation of **5** by a cytochrome P450 monooxygenase FtmC yields desmethyltryprostatin A (**6**), which is further converted to tryprostatin A (**7**) by methyltransferase FtmD. Finally, the second cytochrome P450 monooxygenase FtmE allows **7** to afford **3** via radical-prompted intramolecular cyclization. We cloned the open reading frames of these five genes (*ftmA*–*E*) tandemly connected with the active promoter for expression in *S. cerevisiae*. Additionally, to increase metabolite production, we established an engineered yeast strain SCKW5, a derivative of BY4705 containing two exogenous genes, *matB* (malonyl-CoA synthase from *Rhizobium leguminosarum*) and *npgA* (phosphopantetheinyl transferase from *Aspergillus nidulans*) in the chromosome (Fig. 3a) [23]. We confirmed the production of **4**, **5**, and **6**, whereas **7** and **3** were not observed in this system. Although our attempts toward the mass production of **3**, such as optimization of culture conditions, using highly expressed promoters, and adjusting the codon usage of the genes to that of *S. cerevisiae*, finally provided **3** as a trace amount in the culture, it was insufficient for the subsequent screening of the spiro-forming enzyme. We concluded that the methylation reaction catalyzed by FtmD was not sufficient in this condition, possibly due to either the failure of the production of catalytically active enzyme in the cell or lack of the cofactor *S*-adenosyl-l-methionine (SAM) for secondary metabolism. Therefore, we next exploited the filamentous fungus *Aspergillus niger*, a producer of secondary metabolites containing methyl groups, as a heterologous expression host. We chose *A. niger* A1179 (publicly available from the Fungal Genetics Stock Center, USA), a uridine/uracil auxotrophic strain (*pyrG*-) capable of being transformed by the protoplast-PEG method with a *pyrG* selection marker [24]. We modified the commercially available pPTRII expression plasmid [25] to construct a plasmid for the delivery of five *ftm* genes linked with high expression promoters [26] (the *ftmA–E* genes to the following promoters, *glaA*, *amyB*, *gpdA*, *mbfA*, and *glaA*, respectively). The *A. niger* transformed with this plasmid showed very high productivity of **3** (25 mg L^−1^) under starch-induced expression conditions (Fig. 3b).

**Fig. 4:**
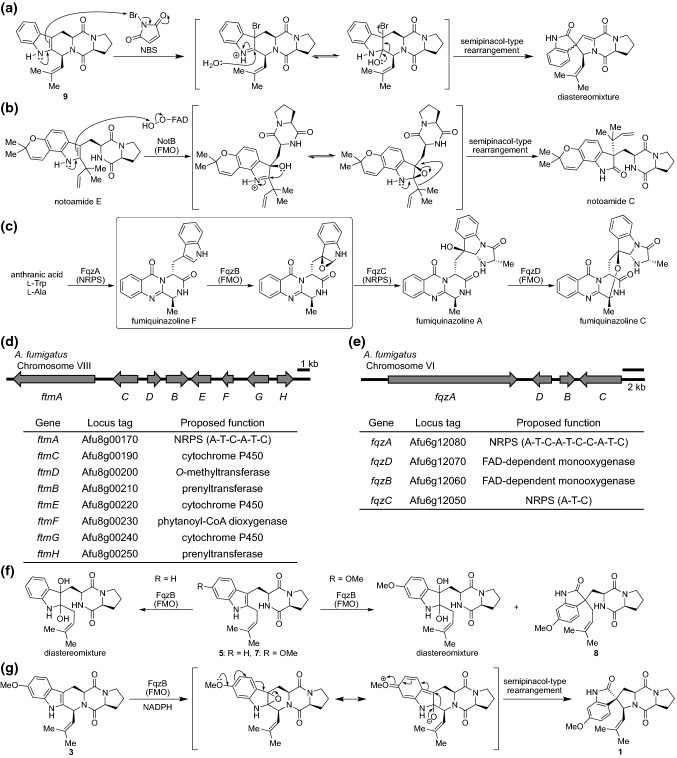
Determination of the spiro-forming enzyme for the biosynthesis of spirotryprostatin A. **a** A biomimetic synthesis for producing spirotryprostatin scaffold. **b** NotB-catalyzed semipinacol-type rearrangement in the biosynthesis of notoamide C. **c** The enzymatic function of FqzB in fumiquinazoline biosynthesis. **d** The BGC of tryprostatin/fumitremorgin. **e** The BGC of fumiquinazolines. **f** In vitro enzymatic reaction of FqzB with tryprostatins A and B. **g** FqzB-catalyzed ortho-quinonemethide formation is the crucial step in the spiro-ring formation

Hence, the situation was set to begin screening of the spiro-forming enzyme. We noticed that “biomimetic total synthesis” could provide valuable insight into how natural products are biosynthesized naturally. For the chemical synthesis of this class of compounds, a crucial step is a semipinacol-type rearrangement via oxidation at the 2,3-position of the indole ring to form spirooxindole using *N*-bromosuccinimide (NBS) [27]. Spiro-ring structures in spirotryprostatins were constructed synthetically using these methods (Fig. 4a). We predicted that an enzyme responsible for the oxidation at the indole ring was likely involved in the biosynthesis of spirotryprostatins. According to a report showing the enzyme NotB that converts notoamide E to notoamide C via the epoxidation of indole and subsequent semipinacol-type rearrangement (Fig. 4b) [28], we looked for a candidate gene that encodes a similar protein to NotB in the genome of *A. fumigatus*. We found *fqzB* (Afu6g12060), which encodes a flavoprotein and has been reported to oxidize an indole ring of fumiquinazoline F to generate a 1,2-epoxyindole intermediate toward the biosynthesis of fumiquinazoline A (Fig. 4c) [29]. Although *fqzB* was located at a distance to the *ftm* cluster (Fig. 4d, e), we sought to verify its role in spirotryprostatin biosynthesis because of the substrate’s shared substructure, the tryptophan residue. We introduced *fqzB* into the previously established plasmid synthesizing **3** in *A. niger* and successfully determined the production of **1** in the culture. The production titer (1.0 mg L^−1^) of the heterologous production was 333-fold higher than that of the wild-type strain *A. fumigatus* BM939 [30].

**Fig. 5:**
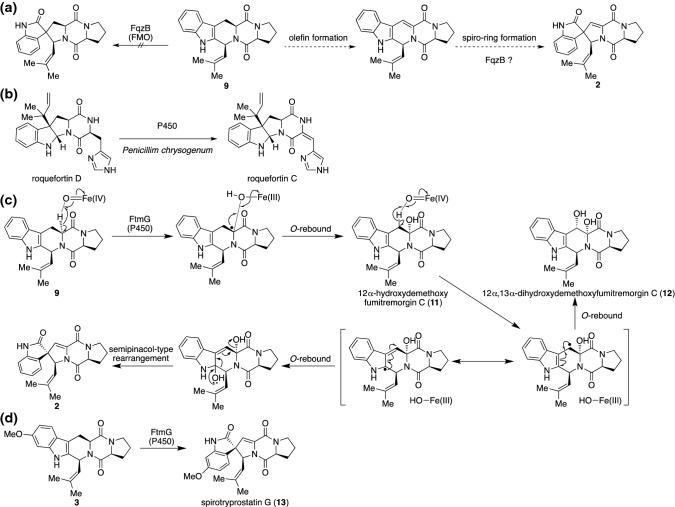
Determination of the spiro-forming enzyme for the biosynthesis of spirotryprostatin B. **a** In vitro analysis revealed the alternative pathway toward spirotryprostatin B. **b** Proposed pathway toward roquefortin C from roquefortin D. **c** Proposed mechanism of FtmG-catalyzed spiro-ring formation in the spirotryprostatin B biosynthesis. **d** FtmG is capable of transforming 3 into spirotryprostatin G (**13**), a new congener in spirotryprostatins

In addition to the in vivo determination, we verified the catalytic activity of FqzB in vitro. While the reaction yield was less than 1.0%, a recombinant FqzB protein obtained from *Escherichia coli* was able to transform **3** into **1** in the presence of NADPH, suggesting that the low reaction rate of the enzyme might have caused the trace production of **1** in the culture of wild-type *A. fumigatus*. It should also be noted that FqzB might be dedicated to the biosynthesis of fumiquinazolines, not to that of spirotryprostatins, due to its occurrence in the fumiquinazoline gene cluster. FqzB was also capable of converting **5** and **7** to the corresponding products by indole oxidation. Interestingly, the 2-oxindole derivative (**8**) that could be converted via semipinacol-type rearrangement was generated from **7** by FqzB (Fig. 4f), indicating that the generation of a para-quinone methide intermediate from the methoxy indole derivative was able to assist the rearrangement (Fig. 4g). Although FqzB showed a promiscuous substrate scope, it did not transformed demethoxylfumitremorgin C (**9**) into a spiro-compound, the predicted precursor of **2**. The structural differences between **1** and **2**, the lack of the methoxy group, and the presence of the unsaturated bond at C-12,13 in **2** led us to predict that the unsaturation reaction would precede the spiro-ring formation in the biosynthesis of **2** (Fig. 5a).

To decipher how the α,β-unsaturated amide was formed in the diketopiperadine (DKP) scaffold of **2**, we investigated for natural products harboring a similar partial structure. We identified that roquefortines originally isolated from the fungus *Penicillium chrysogenum* carry the olefin at the histidine residue of the DKP, and their BGC was reported [31]. Although the enzyme catalyzing olefin formation was not investigated, we predicted each biosynthetic step toward roquefortine C production. We estimated that cytochrome P450 is likely responsible for olefin formation from the α,β-saturated amide because it promotes C–H activation-initiated oxidation (Fig. 5b). With BLASTP searches against the genome of *A. fumigatus* using the P450 gene of the roquefortin gene cluster as a query, we identified a cytochrome P450, FtmG, encoded in the *ftm* gene cluster as the most relevant protein. FtmG has been reported to install a vicinal dihydroxyl group at the C-12,13 of **3** for the formation of 12α,13α-dihydroxyfumitremorgin C (**10**) [22]. However, it has never been investigated as an olefin-forming enzyme. Thus, we tested whether FtmG catalyzes olefin formation using a yeast-based biotransformation assay. Surprisingly, using **9** as a substrate, we confirmed the generation of **2** as well as 12α-hydroxydemethoxyfumitremorgin C (**11**) and 12α,13α-dihydroxydemethoxyfumitremorgin C (**12**) in the culture of *ftmG-*expressing *S. cerevisiae* BY4705 but not in the negative controls (Fig. 5c). This unanticipated result showed us that FtmG, not FqzB, is indeed a spiro-ring-forming enzyme for the production of **2**. The enzymatic reaction of FtmG was further verified by an in vitro assay with the microsome fraction obtained from the yeast transformant expressing *ftmG*. The structures of the reaction products were unambiguously elucidated based on NMR spectroscopy. Accordingly, four genes (*ftmA*, *ftmB*, *ftmE*, and *ftmG*) were introduced into the host *A. niger* for de novo production of **2**, and they successfully afforded **2** with a titer of 4.0 mg L^−1^ (145-fold increase to that of the wild type) in this system. As seen above, the spiro-ring formation of two structurally similar natural products, **1** and **2**, is unexpectedly catalyzed by distinct enzymes, FqzB and FtmG, respectively, with independent reaction mechanisms. For the biosynthesis of **1**, the initial step is the epoxidation of the indole ring catalyzed by the hydroperoxide reaction center of the flavoenzyme FqzB. Then, the electron-rich aromatic methoxide accelerated an epoxide opening concomitant with the formation of a labile ortho-quinone-methide intermediate, which immediately undergoes semipinacol-type rearrangement to afford **1** (Fig. 4g). In contrast, biosynthesis of **2** occurs through a radical mechanism in which the Fe(IV) =O catalytic core in the heme of cytochrome P450 FtmG catalyzes stepwise C–H activation. The crucial step for spirocycle formation is the second round of radical generation at C-13 of **11**. We proposed that the radical migrated from C-13 to C-2 ahead of rebound to the Fe(IV)–OH center. Finally, semipinacol-type rearrangement of the hemiaminal intermediate provides a spiro-ring with the α,β-unsaturated amide scaffold of **2** (Fig. 5c). Thus, we showed the distinct mechanisms, the epoxide route, or the radical route, for the spiro-carbon formation in the spirotryprostatin biosynthesis. The results further extended to produce a novel natural product, spirotryprostatin G (**13**), carrying the aromatic methoxide with α,β-unsaturated amide, by utilizing the biocatalyst FtmG with the substrate **3** (Fig. 5d). We demonstrated that the *A. niger*-based fungal expression system is a powerful tool for identifying the enzyme function and enhancing the production titer for natural products isolated from natural resources as trace components. It should be noted that successive studies have established a modified system using *Aspergillus nidulans* A1145 as a heterologous expression host, capable of harboring triple plasmids, for the study of fungal natural products [32–34].

## Expression of the transcriptional regulator in the silent gene cluster

**Fig. 6:**
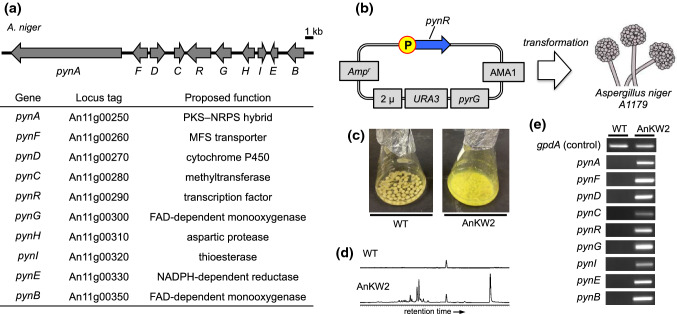
Activation of a silent gene cluster (*pyn* cluster) in *A. niger*. **a** The *pyn* gene cluster for the biosynthesis of pyranonigrin E1. **b** The experimental design for the overexpression of the transcription factor, *pynR*. **c** The culture of the *pynR*-expressed mutant (AnKW2) and the wild type (WT). **d** HPLC analysis of AnKW2 and WT. The chromatogram was monitored at *λ* = 280 nm. **e** RT-PCR analysis of the *pyn* cluster

**Fig. 7:**
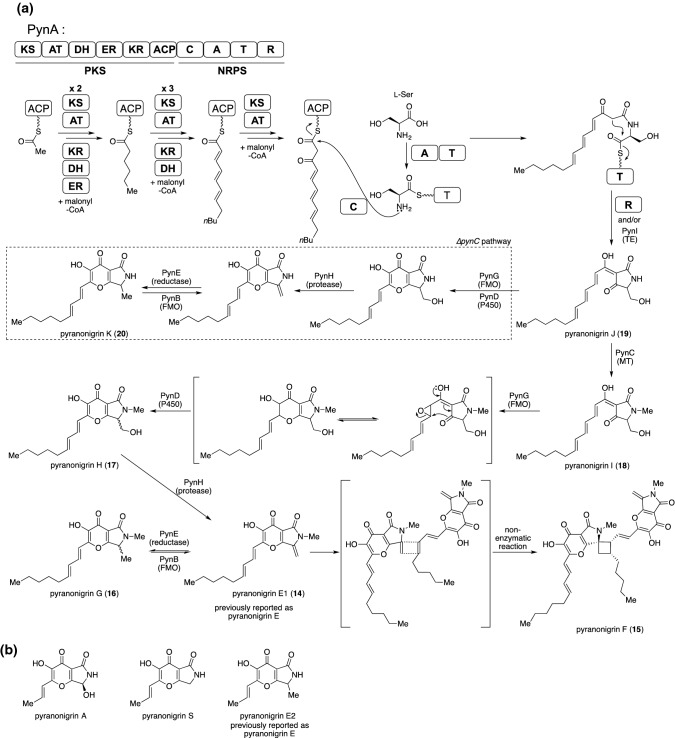
The pyranonigrin biosynthesis. **a** Proposed biosynthetic pathway of pyranonigrin E1, in which production was activated through the overexpression of *pynR*. **b** The chemical structures of the other pyranonigrins

In contrast to the above study, in which biosynthesis of rare natural products was achieved by the overexpression of multiple biosynthetic genes in the heterologous host, we thought that a more rapid, easy-handling, and versatile method needed to be developed. We focused on pathway-specific transcription factor genes that were occasionally located within the BGCs of filamentous fungi [35]. To identify such genes, we exploited a genome mining approach based on the homology search of the desired proteins in the genome database. The transcription factor we examined was the gene embedded in the BGC that was transcriptionally inactive and consisted of a unique combination of auxiliary enzyme genes. We identified *pynR* (An11g00290) in the genome of *A. niger* as a candidate because its BGC that contains a hybrid of polyketide synthase–non-ribosomal peptide synthetase (PKS–NRPS) was thoroughly silent by RNA-seq analysis. In addition, they contained multiple proteins with unknown functions, such as PynH, which harbors a fold of aspartic protease that had never been reported as a modification enzyme for the biosynthesis of natural products (Fig. 6a). Therefore, we employed *A. niger* A1179 to be transformed by the plasmid carrying the *pynR* gene connected to the inducible *glaA* promoter (Fig. 6b). Interestingly, the culture broth exhibited a pale yellow color under the condition of *pynR* expression induction (Fig. 6c). Comparative LC–MS analysis between the wild-type and the mutant revealed a specific production of more than 20 chemical species in the mutant (Fig. 6d). Our RT-PCR analysis indicated that all genes in the *pyn* gene cluster, not only for *pynR*, were strongly transcribed in the *pynR-*expressing mutant (Fig. 6e). Hence, we characterized the produced compounds by purification and following NMR-based structure elucidation, revealing that structurally novel metabolites, pyranonigrins E–G (**14**–**16**), were discovered with high production titers (each more than 20 mg L^−1^, Fig. 7a). Pyranonigrin E is a relative of pyranonigrins A and S, which were also isolated from *A. niger* and have a γ-pyrone ring in the structure (Fig. 7b) [36]. However, the difference in alkyl chain length and amino acid origin suggested that the distinct enzyme might biosynthesize them. Of note, prior to our report, pyranonigrin E was independently discovered and designated by Awakawa et al. [37] and Riko et al., who reported a different chemical structure [38]. Therefore, to avoid confusion, we propose the former and the latter be referred to as pyranonigrin E1 and pyranonigrin E2, respectively. We were further interested in the biosynthesis of pyranonigrins that contain characteristic substructures such as fused γ-pyrone, amino acid-derived *exo*-methylene, and cyclobutane [39].

To analyze how each biosynthetic enzyme participates in the biosynthesis of pyranonigrins, we constructed a line of knockout mutant that was deficient of the gene allocated to the *pyn* cluster. Each knockout strain was produced by homologous recombination with the protoplast-PEG procedure. We identified newly produced metabolites, pyranigrins H–K (**17**–**20**), from the mutants, ∆*pynH*, ∆*pynG*, ∆*pynC*, and ∆*pynB*. The chemical structures of the obtained products suggested the respective enzyme’s role in the biosynthetic pathway, and they were subsequently determined by experiments, such as enzymatic reactions with recombinant proteins or biotransformation assays using heterologous expression systems. Briefly, acetyl-CoA, malonyl-CoA, and l-serine were the starting materials for biosynthesis, and they were assembled to produce a tetramic acid intermediate (**19**) by PynA, which is a PKS–NRPS hybrid megasynthase. After methylation to **19** was performed by PynC with SAM to produce **18**, successive oxidations triggered by PynG (FMO) coupled with PynD (cytochrome P450 monooxygenase) afforded **17** as a γ-pyrone ring-containing product. A protein carrying an aspartic protease fold, PynH, catalyzes the dehydration of l-serine-derived secondary alcohol to produce an *exo*-methylene, a hallmark of **14**. Interestingly, **14** and **16** can be interconverted by the catalysis of PynE (reductase) and PynB (FMO). As similar mutual conversions have also been reported in the biosynthesis of chaetoviridin [40], there might be a shared strategy in fungal metabolite biosynthesis, presumably for effectively conducting a prodrug strategy. Furthermore, we confirmed that the dimeric structure of **15** was sufficiently generated from **14** by spontaneous [2 + 2] electrocyclization, rather than by enzyme catalysis. The identification of **20**, which is the demethylated form of **16,** from the ∆*pynC* mutant indicated that the biosynthetic enzymes PynG, PynD, PynH, and PynE have a promiscuous substrate scope. We showed that it is an effective strategy to provide structurally novel natural products through biosynthetic analyses of metabolites that are encoded by the silent gene cluster of fungus. This strategy was further extended to explore other fungal metabolites such as shanorellins [41] from *Chaetomium globosum* and aspirochlorines [42] from *Aspergillus oryzae*.

## Activation of the biosynthetic pathway in basidiomycete fungi

**Fig. 8:**
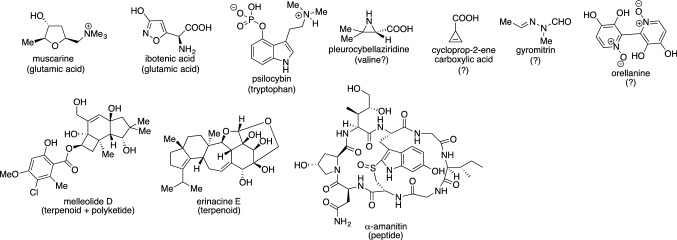
The secondary metabolites isolated from basidiomycete fungi. The proposed biosynthetic origin is bracketed

Previously discussed studies intended to activate specific biosynthetic genes of interest that were bioinformatically predicted to be involved in the biosynthesis of natural products. The following section presents a study aimed to activate nonspecific regions in the genome to acquire unexpected natural products. Similar to ascomycetes, basidiomycetes are one of the microorganisms that make up the fungal kingdom. They are widely known to produce fruiting bodies (mushrooms) for sporulation, and many of them have been used as traditional medicines in the world, while no small numbers of species are toxic to humans [43]. Therefore, basidiomycetes have been recognized as a treasure trove of biologically active substances for the development of drugs. Many natural products have been identified through traditional methods such as picking in wild fields and artificial cultivation. Taking a look at the genomes of basidiomycete fungi, it reveals the natural products’ biosynthetic genes with a domain structure similar to that of ascomycetes. Indeed, polyketides (for example, melleolide D), peptides (for example, α-amanitin), and terpenes (for example, erinacine E), in which biosynthesis is well studied, have also been isolated and structurally determined from basidiomycetes (Fig. 8). Moreover, basidiomycetous natural products that are likely produced through unusual biosynthetic pathways have also been identified, such as muscarine, ibotenic acid [44], psilocybin [45], pleurocybellaziridine [46], cycloprop-2-ene carboxylic acid [47], orellanine [48], and gyromitrin [49] (Fig. 8). Of note, many compounds are known to exhibit potent biological activity, while their biosynthetic machinery has remained elusive.

According to the examples of successful activation of biosynthetic pathways from silent BGCs of ascomycetes, phylogenetically related basidiomycetes are also potential candidates for biosynthesizing hidden natural products encrypted in the genome. However, distinct from ascomycetes, limited numbers of basidiomycetes species have been genetically engineered with knockout and overexpression, hampering the biosynthetic analysis of its natural products. Therefore, to establish a way to study the natural products, we selected the mushroom fungus *Coprinopsis cinerea *[50] (commonly known as gray shag), used as a model organism for studying fruiting body formation with genetics due to its ability to manipulate genes.

**Fig. 9:**
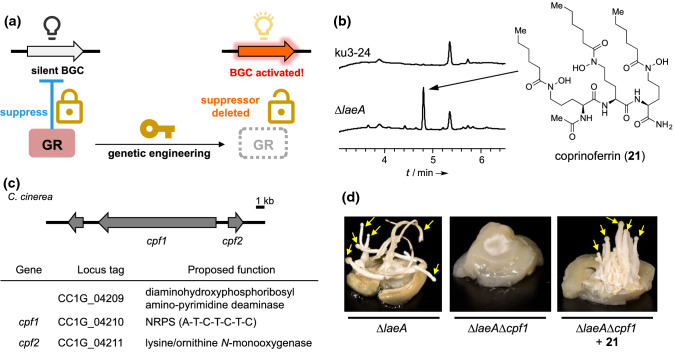
Activation of the cryptic pathway in *C. cinerea*. **a** The strategy for the activation of secondary metabolism by a deletion of the global regulator (GR), *laeA*. **b** HPLC analysis determined the increased production of coprinoferrin in *∆laeA*. The parent strain (ku3-24) also produces **21** with a few production titer. **c** The BGC of coprinoferrin. **d** The fruiting body formation assay. *∆laeA∆cpf1* did not differentiate into the fruiting body. However, the phenotype was restored when **21** was administered to the culture

**Fig. 10:**
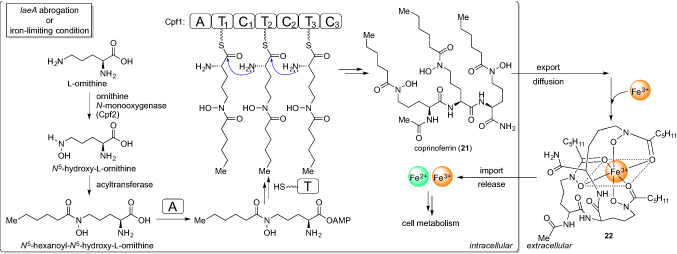
The proposed biosynthetic pathway of coprinoferrin

To activate the cryptic pathway in *C. cinerea*, we focused on *laeA* homologs encoded in the genome. LaeA is a member of the conserved transcriptional regulatory velvet complex involved in secondary metabolism and sexual development in ascomycetes [51]. Because the loss of *laeA* decreases the production of secondary metabolites, LaeA is recognized as a global regulator of secondary metabolism in ascomycetes [52]. While the molecular function of LaeA remains unclear, the encoded protein has an SAM-binding motif that is representative of methyltransferase, and is predicted to be involved in epigenetic regulation associated with secondary metabolism [53]. Therefore, we designed to exploit a *laeA* homologous gene in *C. cinerea* to induce the production of natural products with novel chemical structures. We selected a gene (CC1G_00498, hereafter *laeA*) homologous to *laeA* from *A. fumigatus* as a candidate (identity = 48%). We engineered *C. cinerea* ku3-24 strain [54], which was deficient in *ku70* for adequate gene replacement, to construct the *laeA* disruption mutant (*C. cinerea ∆laeA*) and analyzed the production of the metabolites (Fig. 9a). We observed an increased production of a compound with the molecular formula C_35_H_65_N_7_O_10_ by LC–HRESIMS analysis in the ∆*laeA* strain (Fig. 9b). The compound was purified from the large-scale cultivation of ∆*laeA*, structurally elucidated to be an unidentified molecule, and designated as coprinoferrin (**21**) [55]. The chemical structure of **21**, which carries a trimeric *N*^5^-acylated-*N*^5^-hydroxyl-l-ornithine residue, suggested that it might form a complex with Fe(III) or other metal ions [56]. We experimentally confirmed the Fe(III)-binding property of **21**, and its iron complex (**22**) was also detectable in the broth of ∆*laeA* culture. It has been suggested that **21** plays the role of siderophore [57] widely used for incorporating environmental Fe(III) into the cell in various microorganisms. To determine the contribution of **21** to hyphal growth and development in *C. cinerea*, we analyzed the biosynthesis of **21**. Based on its chemical structure, we searched the genome of *C. cinerea* and identified CC1G_04210 (hereafter *cpf1*) that encodes NRPS carrying a single adenylation module with triplicated thiolation-condensation didomains, as a candidate gene (Fig. 9c). Gene knockout experiments confirmed that *cpf1* is essential for the production of both **21** and **22**. Additionally, characteristic phenotypes such as stunning hyphal growth and deficiency in fruiting body formation were observed in the *C. cinerea ∆laeA∆cpf1* strain, while these phenotypes were restored by administration of **21** to the culture medium (Fig. 9d). These results suggest that *C. cinerea* biosynthesizes **21** to effectively acquire environmental Fe(III) and utilize it for their growth and development. Additional experiments determined that *C. cinerea ∆laeA∆cpf1* was able to form a fruiting body where excess Fe(III) was accumulated, but **21** did not require a high concentration of Fe(III). This suggests that **21** is able to diffuse into the medium and solubilize and transport Fe(III) into the fungal cells (Fig. 10). We also observed trace production of **21** in the wild type *C. cinerea*, whereas, the maximum titer was tenfold lower than that of the *∆laeA* strain. Although the reason for the higher accumulation of **21** in *C. cinerea* in response to *laeA* deficiency remains unexplained, we showed that genetic engineering of the global regulator is a strategy for the activation of secondary metabolism in basidiomycete fungi. Interestingly, the gene cluster containing *cpf1* is highly conserved in more than two hundred genome-sequenced basidiomycete fungi. We have recently confirmed that some of the species also produce **21**, suggesting that **21** may be a common metabolite among a kind of basidiomycetes, and it is possible to have a role in driving the growth and development of mushroom fungi. Although many fungi produced **21**, our strategy enabled the discovery of the molecule for the first time. As such, we showed that genetic engineering is a powerful tool for the discovery of natural products.

## Closing remarks

In this review, we summarized our recent studies focusing on the activation of biosynthetic pathways in filamentous fungi. We introduced yeast- and *A. niger*-based heterologous expression systems for the sustainable production of natural products that were limited to the original fungal strain. This synthetic biology strategy was also demonstrated to decipher the specialized biosynthetic pathways of natural products. However, there are still issues to be addressed in these systems. In particular, in the yeast system, we could not obtain the desired products due to several unknown factors. Although we raised some possibilities, such as the loss of enzyme activity in the heterologous host or insufficient supply of the cofactors, we realized that it is not always easy to determine the reasons for the intercepting metabolic process. A systematic understanding of heterologous cells, such as the balance of gene expression, substrate supply level, solubility, or folding of the produced enzyme, will provide a versatile tool for the metabolic engineering of natural products. Second, we showed the genetic engineering of the transcription regulator embedded in the silent BGC of fungus to unveil novel natural products with high titers. In combination with the pathway disruption by knockout strategy, it further allowed us to identify a series of biosynthetic products and provide information on the pathway toward the final product from the starting materials. Subsequent analyses revealed unique biosynthetic systems, including enzymatic function, which catalyze an unprecedented biochemical transformation. Finally, our recent strategy to activate the basidiomycetous biosynthetic pathway was introduced. Genetic engineering of a global regulator *laeA* in *C. cinerea* was capable of inducing siderophore biosynthesis. Our reverse genetic approach based on the deletion of *cpf1* responsible for coprinoferrin production revealed the significant roles of siderophore in mushroom fungus on hyphal growth and fruiting body formation. Although this strategy does not apply to a wide range of basidiomycete fungi due to the difficulty in genetically transforming them, recent technological advances such as genome editing [58] will help decipher the secondary metabolism of basidiomycetes. In addition, one of our current missions is to use *C. cinerea* as a heterologous host for the expression of the gene from basidiomycete, and we hope it will be achieved in the near future [59]. In summary, we insist that it is still possible to provide structurally novel secondary metabolites, as there is a vast amount of accumulated information on biosynthetic genes in the database. These valuable resources, obtained worldwide, need to be efficiently utilized for drug discovery and natural product research.
